# Developing a validated methodology for identifying clozapine treatment periods in electronic health records

**DOI:** 10.1186/s12888-024-06022-5

**Published:** 2024-08-27

**Authors:** Aviv Segev, Risha Govind, Ebenezer Oloyede, Hamilton Morrin, Amelia Jewell, Rowena Jones, Laura Mangiaterra, Stefano Bonora, Ehtesham Iqbal, Robert Stewart, Matthew Broadbent, James H. MacCabe

**Affiliations:** 1 NIHR Biomedical Research Centre, South London and Maudsley NHS Foundation Trust, London, UK; 2 Department of Psychosis Studies, Institute of Psychiatry, Psychology and Neuroscience, King’s College London, London, UK; 3 Faculty of Medicine, Tel Aviv University, Tel Aviv, Israel; 4 Shalvata Mental Center, Hod Hasharon, Israel; 5 Institute of Psychiatry, Psychology and Neuroscience, King’s College London, London, UK; 6 Pharmacy Department, South London and Maudsley NHS Foundation Trust, London, UK; 7 Department of Psychiatry, University of Oxford, Oxford, UK; 8 Maudsley Training Programme, South London and Maudsley NHS Foundation Trust, London, UK; 9 Birmingham and Solihull Mental Health Foundation Trust, Birmingham, UK; 10 Institute for Mental Health, University of Birmingham, Birmingham, UK; 11 Atkinson Morley Regional Neurosciences Centre, St. George’s Hospital, London, UK; 12 Department of Health Sciences, Università Degli Studi Di Milano, Milan, Italy; 13 National Psychosis Unit, South London and Maudsley NHS Trust, Beckenham, Kent, UK

**Keywords:** Clozapine, Schizophrenia, Psychosis, Databases, Algorithm, CRIS, Maudsley, EHR

## Abstract

**Background:**

Clozapine is the only recommended antipsychotic medication for individuals diagnosed with treatment-resistant schizophrenia. Unfortunately, its wider use is hindered by several possible adverse effects, some of which are rare but potentially life threatening. As such, there is a growing interest in studying clozapine use and safety in routinely collected healthcare data. However, previous attempts to characterise clozapine treatment have had low accuracy.

**Aim:**

To develop a methodology for identifying clozapine treatment dates by combining several data sources and implement this on a large clinical database.

**Methods:**

Non-identifiable electronic health records from a large mental health provider in London and a linked database from a national clozapine blood monitoring service were used to obtain information regarding patients' clozapine treatment status, blood tests and pharmacy dispensing records. A rule-based algorithm was developed to determine the dates of starting and stopping treatment based on these data, and more than 10% of the outcomes were validated by manual review of de-identified case note text.

**Results:**

A total of 3,212 possible clozapine treatment periods were identified, of which 425 (13.2%) were excluded due to insufficient data to verify clozapine administration. Of the 2,787 treatments remaining, 1,902 (68.2%) had an identified start-date. On evaluation, the algorithm identified treatments with 96.4% accuracy; start dates were 96.2% accurate within 15 days, and end dates were 85.1% accurate within 30 days.

**Conclusions:**

The algorithm produced a reliable database of clozapine treatment periods. Beyond underpinning future observational clozapine studies, we envisage it will facilitate similar implementations on additional large clinical databases worldwide.

**Supplementary Information:**

The online version contains supplementary material available at 10.1186/s12888-024-06022-5.

## Introduction

Treatment-resistant schizophrenia (TRS) is associated with poor prognosis, long-term disability, and increased mortality [1]. The introduction of clozapine in the late 1950s provided clinicians with a unique option in the pharmacological treatment of individuals with TRS [2, 3]. Despite its discovery many decades ago and the development of many drugs since then, clozapine remains the treatment of choice in TRS due to its superior efficacy [4]. Current evidence indicates that of the 30% of patients diagnosed with schizophrenia who do not respond to conventional antipsychotics, 50% will respond to clozapine [5]. Moreover, several studies have shown that clozapine yields the best prognosis versus other antipsychotics, not only for psychiatric clinical scales but also for broader health outcomes, including all-cause mortality [6–9].

Unfortunately, despite a considerable evidence-base for therapeutic benefits, clozapine is associated with a range of adverse effects, including potentially life-threatening events such as myocarditis, ileus and blood dyscrasias, mandating regular blood tests [10, 11]. As such, there has been much interest in the study of clozapine: basic-science research, that attempts to elucidate the reasons for its superior efficacy or the mechanisms underlying its side effects [12], clinical and laboratory biomarkers to predict its efficacy [13] and clinical studies to better understand, detect and manage its adverse events [14, 15]. Such insights may help to diminish underutilization of clozapine [16] and to prevent unnecessary clozapine cessation and the associated increased risk of relapse [17]. Many of these clinical observational studies rely on small, biased samples, and as such are disadvantaged by low statistical power and uncertainty around the generalizability of findings. In view of this, it is important to enable investigators to reliably study large, unbiased cohorts of patients prescribed clozapine, with accurate data on the dates when treatment was started and stopped.

South London and Maudsley NHS Foundation Trust (SLaM) is one of the largest mental health providers in Europe, catering to all secondary mental health care needs of over 1.3 million people spanning four London boroughs (Lambeth, Southwark, Lewisham, and Croydon). It contains clinical records of over 500,000 patients, including many individuals diagnosed with psychotic spectrum disorders, and patients who were or are currently prescribed clozapine. In the 2000s, SLaM records became digital and complete electronic health records (EHR) became available during 2006. In 2008, data from the SLaM EHR were made available to researchers through the Clinical Record Interactive Search (CRIS), which is a de-identified copy of the entire SLaM EHR [18]. The granularity of this type of data resource presents valuable opportunities for novel and informative observational studies. However, as with all real-world databases, there is the potential for input errors or missing data. Therefore, when using such data for research, data cleaning, validating, and processing of the desired cohort are required. Overcoming these challenges for clozapine pharmacoepidemiology requires a collaboration of clinicians, familiar with the patterns and protocols surrounding the usage of the medication, alongside informaticians, proficient in handling and analysing real-world big data. This paper describes the rationale, process and heuristics-based algorithms used to create a database of clozapine treatment periods, derived from CRIS at SLaM, to serve as a resource for large-scale retrospective clozapine studies. The generation of this database provides great potential for upcoming observational studies on clozapine. Beyond enabling studies on SLaM users, the heuristics and algorithms outlined in this paper can be adapted, with appropriate modifications, to suit any other extensive clinical database resembling CRIS in terms of data sources on an international scale. Consequently, it will facilitate the development of additional databases on clozapine treatment periods, thereby laying the groundwork for further research in diverse countries and psychiatric services.

## Methods

### The data sources – CRIS and ZTAS databases

The Clinical Record Interactive Search (CRIS), previously described, makes available all SLaM electronic health records for secondary analysis within a robust data security and governance framework [19].

The Zaponex Treatment Access System (ZTAS) is one of three mandatory blood monitoring service providers in the UK. All patients prescribed clozapine at SLaM are registered with ZTAS [20]. ZTAS has a database of all the mandatory blood test results and all the clozapine treatment-related statuses (e.g., on-treatment, discontinued etc.) assigned to each patient.

SLaM’s Clinical Data Linkage Service (CDLS) provides a secure data environment that allows CRIS to be linked with other external clinical and non-clinical databases, including ZTAS data, using individual matching but then discarding the identifiers, allowing the data to be made available in the same de-identified format as CRIS [21].

The linkage between CRIS and ZTAS, facilitated through CLDS, is the foundation of this cohort. The two databases were first linked in May 2016, followed by a refresh in October 2019. Therefore, the time frame for the current study starts with the establishment of ZTAS in 2004, and ends with its most recent linkage to CRIS, in October 2019.

### Clinical aspects of clozapine prescription

There are several aspects of clozapine treatment that make it challenging to determine if and when clozapine treatments begin and end from the aforementioned databases. In clinical practice, there is often extensive discussion with the patient and treating team regarding the possibility of starting clozapine, for months or even years before the treatment is started. Thus, relying on natural language processing tools, which have shown success in identifying medications through textual references in medical records, may result in numerous false positives, particularly in the case of clozapine.

Patients may have single or multiple periods of clozapine treatment. Due to the adverse-effects profile of clozapine and its mandatory monitoring, any cessation of clozapine lasting more than 48 h requires re-initiation of the drug and blood monitoring as though for the first time [22]. Our algorithm aimed to identify each clozapine treatment period, even when several were recorded for the same patient. This was further complicated by the fact that patients may be prescribed clozapine for long periods but with infrequent clinical contacts, so the algorithm must infer whether there was a treatment break between two clinical contacts.

Another complication is that patients are sometimes registered with ZTAS but are ultimately not prescribed clozapine for various reasons (e.g., non-adherence, medical contraindications), or there may be a long delay between registration and receipt of the first dose.

### Outline of algorithm

The first step was designed to confirm the validity of the treatment period, meaning that clozapine was indeed administered, rather than just intended to be prescribed. In addition, data were collected to define each treatment period, which involved identifying start- and end-dates. At the second stage, we used data from adjacent periods to further confirm clozapine administration, and to determine when two apparently separated treatments, were in fact one continuous treatment. Three data sources were used for this purpose (described in detail below): i) patients' recorded status, ii) blood test monitoring records, and iii) pharmacy dispensing records.

When devising the algorithm, it was decided to value precision over recall. Thus, the algorithm takes a conservative approach, even at the expense of missing potential treatment periods.

As part of the algorithm development, each heuristic implemented in this algorithm was examined separately. However, the validation and verification of the entire algorithm was done as a whole.

### First data source – patient status

In clinical practice, registration with ZTAS is required for clozapine to be dispensed and administered. ZTAS receives notification and grants approval for each initiation of clozapine. When a clozapine treatment ends, the hospital pharmacy will report it to the ZTAS team, and if an additional clozapine treatment attempt is planned, re-registration with ZTAS is required. Possible patient statuses include "on-treatment", "interrupted", "discontinued", "transferred" or "non-rechallengeable" (and several variations of these). A patient’s status changes over time, and the dates of change are recorded, thus a history of dated status changes is stored. Thus, the status of the patient appears at face value to be a relatively robust and reliable dataset.

However, status was found to be inconsistently recorded in practice: some patients had multiple "on-treatment" entries, or multiple redundant "discontinuation" entries, or a confusing sequence of statuses. For example, if a patient's blood test returns with abnormal results, often a status of "interrupted" would appear on that day, as clozapine administration is paused. If an additional abnormal result re-occurs the following day, the patient's status would change to "discontinued". On the same day, or within a few days, usually after consultation between the ZTAS and clinical teams, the status would then change to "discontinued – final", and then "non-re-challengeable". As a result, each clozapine treatment period could be surrounded by many redundant and sometimes contradictory status entries. Accordingly, we classified all possible statuses to one of two groups – start-signals (e.g., "on-treatment") or stop-signals (e.g., "discontinued").

To overcome the problem of multiple and redundant entries, clozapine treatments were initially identified by locating the first start-signal status (per date), either as the first entry for the patient, or following a previous stop-signal. In the same manner, the end of the treatment was identified as the first stop-signal after a previous start-signal. Stop-signals were ignored if on the same day there was an additional start-signal, during an ongoing clozapine treatment period. The periods between the start- and stop-signals were defined as "tentative clozapine treatment periods", that need to be validated and examined. Tentative treatment periods of less than 7 days were excluded from the analysis. The rational for this exclusion stemmed from several reasons: it is likely that such very short treatment periods would not be significant to the study of clozapine; such a short window of treatment is more likely to represent the intention to administer clozapine, without the patient starting the treatment (or taking very few doses); and difficulty to identify markers for an automated verification for clozapine being administered.

There were several reasons why the start-signals and stop-signals could not be considered reliable on their own. Though the start-signals were designed to be assigned at the start of clozapine initiation, relying on patient status had limitations. Patients who were prescribed clozapine prior to the start date of the ZTAS database at our disposal, and who therefore were added to ZTAS during their clozapine treatment, had an inaccurate "start-signal". Similar problems occurred with patients who were registered for SLaM care after a transfer from another Trust in the UK or a different country whilst already receiving clozapine treatment. Another limitation was that the start-signal was an indicator of ZTAS approving a patient's clozapine treatment but did not necessarily indicate that the clozapine treatment was initiated. Delays in clozapine initiation could stem from different reasons, such as a patient’s refusal, physical deterioration, improvement in mental status, etc., and the actual commencement of clozapine dose titration might start weeks after a start-signal appeared in the status field. While clozapine treatment occurred outside the windows defined by the patient start- and stop-signals only in specific circumstances (described later), the presence of the window did not guarantee that clozapine was in fact administered, or that the start-signal corresponded to the actual administration start-date.

Another caveat was clerical errors of omission or commission. Errors of omission were particularly abundant in older patient records, where recording was less systematic. In such cases, a treatment could be evident in the clinical notes but have no preceding start-signal and therefore potentially missed in an algorithm relying on this. Errors of commission included incorrect status entries recorded. An example was a status entry of "transferred", despite the patient's records clearly showing that they remained under the care of SLaM, or "interrupted" despite the clinical records not indicating any problem or change in clozapine administration. Due to these limitations, it was necessary to address and integrate additional datasets.

### Second data source – blood test monitoring

Blood monitoring information was used both for confirming the authenticity of the treatment period and for re-affirming actual start-dates. For each tentative clozapine treatment period, we established the pattern of blood test monitoring. To identify these patterns, we relied on the UK mandatory monitoring guidelines, which require weekly blood monitoring for 18 weeks, followed by fortnightly monitoring for an additional 34 weeks, after which monitoring is reduced to a monthly basis until the treatment is stopped [22]. Using the timing of blood tests, we aimed to identify several possible patterns of monitoring, with the following hierarchy: (1) Sustained weekly pattern (longer than 5 weeks); (2) Short weekly pattern (5 weeks or shorter); (3) Monthly pattern (of over 6 months); and (4) No pattern. The detailed criteria are elaborated in the supplementary material (S1).

ZTAS contains the results of blood tests and the date they were taken, but also the type of blood test in relation to the clozapine treatment period. The blood test that precedes actual administration is defined as "Baseline" (required for ZTAS approval of clozapine treatment). Tests during the clozapine treatment period are named "New". Tests that were entered retrospectively are defined as "Historical". Therefore, we used this information to further verify the actual start-date of the treatment period. When a "Baseline" blood test was recorded ± 10 days from a start-signal, it was regarded as re-affirming the actual start-date (as opposed to artificial start that, a label given to those starting clozapine prior to 2004 or having started this elsewhere prior to being transferred to SLaM).

Blood test monitoring records, when present, were considered a robust source of information. However, several caveats needed to be taken into consideration. The recording of blood tests in ZTAS did not systematically start with the establishment of ZTAS, and for several years was inconsistent between different service providers within SLaM. As such, a lack of blood test records did not mean blood was not taken. An additional problem was that the type of blood test was recorded improperly at times, and a "baseline" test might be labelled as "new". A third problem, limiting our ability to rely on blood test monitoring, was that blood could be drawn several times prior to clozapine initiation (due to the patient changing their mind about treatment, problematic results, etc.), or after clozapine cessation (mainly when attempting to verify neutrophil count normalization following neutropenia, per UK mandatory guidelines). However, the presence of blood test results outside the "treatment window" of start–end signals (as derived from a patient status) would help to detect errors of omission or commission in status records. A common example was lack of status for patients who were entered into ZTAS in the early days of the system when clerical errors were more likely to occur. In these cases, months and even years of repeated blood tests preceded the first status record. Therefore, in cases where blood tests were recorded more than three times prior to the first status, a new tentative "pre-status" treatment period was defined (though start and stop signals were missing). The reason for omitting cases with three or less blood tests was that it was common to see preparatory blood-results before commencing clozapine, preceding the start signal. In addition, analysing the authenticity of these very short periods was extremely challenging. On the other hand, even if those three blood tests represented part of a genuine clozapine treatment period, the inferred treatment period would not have been underestimated substantially. The pre-treatment period was defined as starting at the first blood test and ending at the last blood test preceding the first start-signal. Additional use of blood-monitoring was conducted to ascertain redundant stop signals. As per UK clozapine protocols, clozapine cessation should be followed by four follow-up blood tests. As such, 5 blood tests or less post a stop-signal was considered a per-protocol follow-up. Instances where more than five blood tests were identified outside of a tentative period were flagged and examined manually. Since there were only three such occurrences, it was determined that developing a specific algorithm to analyse these cases would be unnecessary, and thus they were disregarded.

### Third data source – pharmacy dispensary records

SLaM Pharmacy records of clozapine dispensing, as with all other medications, are incorporated in the CRIS database and are completed both for inpatients and outpatients. Again, these had face validity as an ideal indication of clozapine administration; however, as with other data resources, they came with several caveats. Pharmacy records began inconsistently during the first years of wider SLaM record digitalization and records were consequently often missing in the first years of CRIS. Moreover, records were sometimes omitted due to technical or human errors. Conversely, dispensary records may exist even in cases where the patient did not receive the prescribed medication, often attributable to reasons such as patient non-compliance, although not limited to this factor alone. Therefore, the dispensary records could only serve as supporting evidence and were not sufficient to be used alone. We regarded dispensary records as re-affirming when at least 3 records of clozapine were recorded at 3 different dates, as a single dispensation might occur when a clozapine treatment did not commence (for example, due to patient reluctance).

### Combining the three datasets

**Fig. 1:**
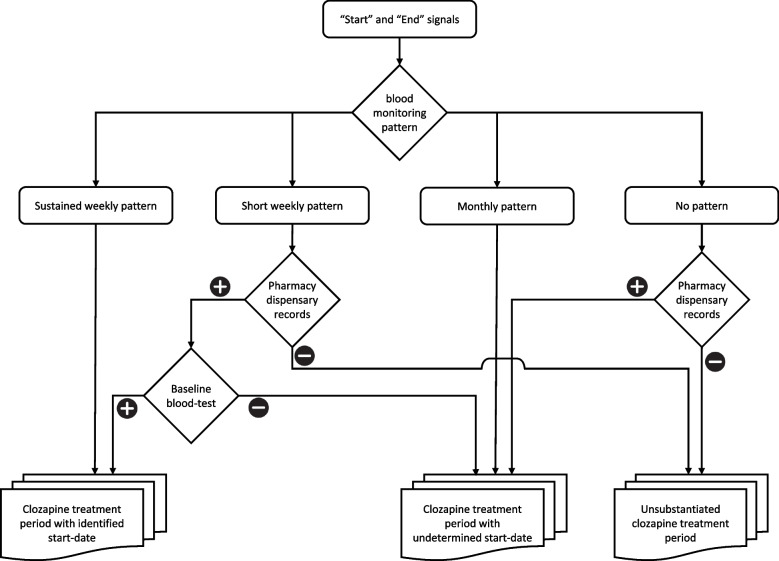
Clozapine treatment periods categorization per blood tests pattern, baseline type blood tests and pharmacy records

Using the described tiers of information, we devised an algorithm (Fig. 1) that classified each tentative clozapine treatment period into one of three possible categories:

1. *Clozapine treatment period with identified start-date* – in which we could have high certainty both that clozapine was administered and that the inferred start-date was a reliable one.
2. *Clozapine treatment period with undetermined start-date* – in which we had enough data to verify, with high level of certainty, that the patient did indeed receive clozapine, but a reliable start-date could not be established. For these treatment periods, there was no valid start-date, but rather a first known date of the treatment. These treatments could have been started only a few days before the first known date or, alternatively, years before it.
3. *Unsubstantiated* – In which there were insufficient data to ascertain that clozapine was given.

Of note, "pre-status" tentative treatment periods that were discovered and defined by blood tests records (i.e., treatment periods that were not created originally from start and end signals) were addressed during the categorization process in the same manner as status-based treatment periods.

### Refining start-dates and end-dates

After the initial classification of treatment periods to these three categories, we implemented further rules to refine the start and end-date of each treatment period, using the treatment period's classification. These refinement rules were created to improve the accuracy of the start-date and the end-date, to overcome clerical errors in the early days of ZTAS, and to improve categorisation. The refinement rules are elaborated in the supplementary material (S2).

### Merging clozapine treatment periods

After each treatment period was assigned a category and the start-date and end-date were refined, more information on treatment periods could be inferred using the adjacent periods. We examined the already-existing datasets, alongside the gaps between each period, in order to identify treatment periods that were wrongly identified by the algorithm as two separate periods. We have used clinical heuristics to merge periods, for example:

1. A clozapine treatment with an identified start-date cannot be a continuation of a previous period, unless it was truncated due to technical error, mandating the gap between the periods to be extremely short, and the first period to be relatively short.
2. Very short gaps between treatment periods (< 7 days) that do not entail even a short weekly pattern in the following treatment period (sometimes referred as "interrupted protocol"), are unlikely a new period and more often represent an error of commission or technical glitch. A common example would be a patient travelling abroad for more than 30 days and forgetting to send blood results. An "interrupted" status would then be added. Upon the patient's return, if clozapine was administered continuously, the status will change again to "on-treatment", representing the same treatment period, assigned for merging. If the patient stopped clozapine while abroad, the algorithm would recognize the start of a new weekly pattern of blood tests, thus categorizing the new treatment period as one with an identified start-date that would not be merged.

The complete set of rules by which treatment periods were merged are outlined in the supplementary material (S3).

### Excluding clozapine treatment periods

Following the merging stage, unsubstantiated treatment periods that were not merged were excluded, and were disregarded in further analyses (except validation). Due to the various indications used, those periods were suspected to be "empty" treatment, meaning that no clozapine was given. These periods remained in the database, unlike the omitted periods, for two reasons:

1. They might be a focus of interest – for example, concerning the reasons that prevented the administration of clozapine.
2. Though suspected to be "empty", this impression relied mainly on the continuing lack of corroborative evidence. However, it was assumed that despite the absence of data, some were false negative treatments, i.e. clozapine was given.

### Validation of results

After forming the new merged table of all clozapine treatment periods, at least 10% of the treatment periods were randomly selected and manually compared to their full text HER records by an experienced psychiatrist (AS). The accuracy of the start-date, end-date and the classification of the treatment periods were manually verified.

## Results

According to the ZTAS database, 2,056 SLaM patients were registered with ZTAS, 41 of whom had never had a blood test or an assigned status. ZTAS recorded 210,173 blood tests and 10,923 statuses for the remaining 2,015 SLaM patients. Patients had a mean of 103 blood tests (SD 76.6, range 1–341), 5 statuses (SD 4.8, range 1–41), and 108 pharmacy dispensaries (SD 98.3, range 1–571).

**Fig. 2:**
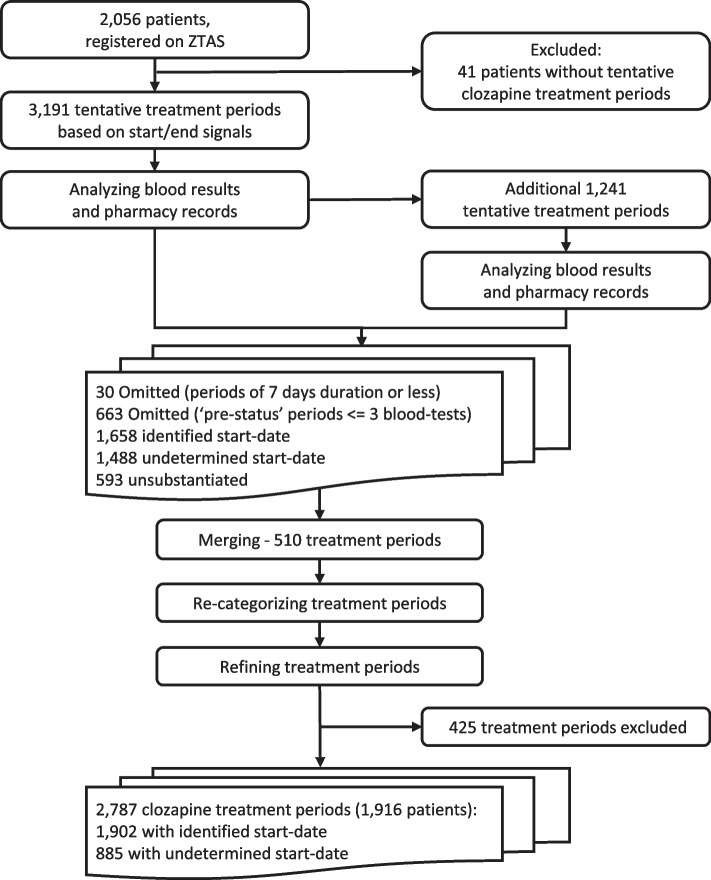
Results of categorization process, per each algorithmic step

Figure 2 shows that 3,191 tentative treatment periods were first identified based on the start- and end-signals. An additional 1,241 tentative periods were identified after analysing the blood test data and pharmacy dispensary data. 693 tentative treatments were omitted; 30 (0.9%) were omitted because the period ended within 7 days, and 663 (53.4%) because they were "pre-status" periods with three or fewer blood tests. 510 tentative treatment periods were merged with adjacent periods based on the criteria outlined in Table S3. Glossary of main definitions is listed in Table 1.

**Table 1:** Glossary of main definitions

**Start-signal** – Assignment of a status marking the start of a clozapine treatment period
**End-signal**—Assignment of a status marking the end of a clozapine treatment period
**Tentative treatment period** – a period between a start- and an end-signal, therefore a potential clozapine treatment
**Pre-status treatment period** – tentative clozapine treatment periods, that were defined despite not having a start-signal, based on the existence of blood-tests, preceding the first status assignment
**Identified start-date** – a first known date of clozapine administration for a specific treatment period that marks a new clozapine initiation with high level of certainty, based on blood tests indicators
**Undetermined start-date**—a first known date of clozapine administration for a specific treatment period, where it is unclear whether this marks a new clozapine initiation or is the first known date of clozapine treatment that was initiated prior to that date
**Unsubstantiated clozapine treatment periods** – periods suspected to contain no clozapine administration

After merging, re-categorizing, and refining, there were 3,212 treatment periods. Of these, 425 (13.2%) remained unsubstantiated treatment periods due to insufficient data to confirm that clozapine was given, and therefore were excluded.

In total, the algorithm identified 2,787 clozapine treatment periods: 1,902 (68.2%) with an identified start-date and 885 (31.8%) with an indeterminate start-date.

The 2,787 treatments belonged to 1,916 patients. The mean number of treatment periods per patient was 1.45 (median 1, interquartile range 1). 1,346 (70.0%) patients had only one treatment period, 373 (19.5%) had two, and 197 (10.3%) had 3–8 periods. 65.6% of the patients were male, 45.3% were of White ethnicity, and 41.7% were of Black ethnicity. The mean age at the point of the first known clozapine treatment was 39.0 (SD 12.1). Demographic characteristics of patients and treatment periods are displayed in Table 2.

**Table 2:** Demographic characteristics per clozapine patients and per treatment periods

Per Patients			
	All Patients *n* = 1,916	Patients who have treatment periods with identified start-dates^a^ *n* = 1,391	Patients who have treatment periods with undetermined start-dates^a^ *n* = 790
Gender			
Male (%)	1,257 (65.6%)	898 (64.6%)	541 (68.5%)
Female (%)	659 (34.4%)	493 (35.4%)	249 (31.5%)
Ethnicity			
White (%)	868 (45.3%)	591 (42.5%)	393 (49.7%)
Black (%)	799 (41.7%)	616 (44.3%)	306 (38.7%)
Asian (%)	150 (7.8%)	111 (8.0%)	55 (7.0%)
Other and unknown (%)	99 (5.2%)	73 (5.3%)	36 (4.6%)
Age at clozapine treatment			
Age at first period, mean (SD)	-	38.7 (12.7)	-
Age at first known period, mean (SD)	39.0 (12.1)	-	39.8 (10.8)
Per Clozapine Treatment Period			
	All treatment periods *n* = 2,787	treatment periods with identified start-dates *n* = 1,902	treatment periods with undetermined start-dates *n* = 885
Gender			
Male (%)	1,821 (65.3%)	1,213 (63.8%)	608 (68.7%)
Female (%)	966 (34.6%)	689 (36.2%)	277 (31.3%)
Ethnicity			
White (%)	1,209 (43.4%)	782 (41.1%)	427 (48.2%)
Black (%)	1,220 (43.8%)	872 (45.8%)	348 (28.0%)
Asian (%)	219 (7.9%)	154 (8.1%)	65 (7.3%)
Other and unknown (%)	139 (5%)	94 (4.9%)	45 (5.1%)
Age at clozapine treatment periods			
Age range at start of period	12–88	12–88	12–76
Periods starting at ages 0–19 (%)^b^	66 (2.4%)	50 (2.6%)	16 (1.8%)
Periods starting at ages 20–39 (%)^b^	1,432 (51.4%)	1,007 (52.9%)	425 (48.0%)
Periods starting at ages 40–59 (%)^b^	1,126 (40.4%)	722 (38.0%)	404 (45.6%)
Periods starting at age 60 and over (%)^b^	163 (5.8%)	123 (6.5%)	40 (4.5%)
Duration of clozapine treatment periods			
Average duration, years	4.5 (4.8)	3.1 (3.7)	-
Median duration (Interquartile range), years	2.2 (7.3)	1.4 (4.4)	-
Shorter than 6 months (%)	-	557 (29.3%)	-
Longer than 2 years (%)	1,437 (51.6%)	815 (42.8%)	622 (70.3%)

^a^Patients can have both periods with identified start-date, and (additional) periods with undetermined start-date, therefore can appear in both columns

^b^The age stratification was done per treatment period, meaning that the same patient can contribute to more than one category of age, per the age in which the treatment period was started

The final result of the algorithm was 2,787 clozapine treatment periods, which belonged to one of two categories: treatment periods with identified start dates and treatment periods with undetermined start-dates. Similar to the two types of start-points, the endpoints can also be categorised into two types: treatment periods with identified end-dates and treatment periods with undetermined end-dates. Treatments with identified end-dates are those ending with a clear end signal, for example, a "discontinued" status or the end of blood monitoring. Treatments with undetermined end-dates result from unavailable information due to patients being transferred outside of SLaM or treatments that remained ongoing at the end of the study window (October 2019). The proportions of treatments with each start-point and endpoint type are elaborated in Table 3.

**Table 3:** Clozapine treatment periods classification per start-point and end-point type

	**Start-date identified**	**Start-date undetermined**	**Total**
End-date identified	936 (33.6%)	369 (13.2%)	1,305 (46.8%)
End-date undetermined	966 (34.7%)	516 (18.5%)	1,482 (53.2%)
Total	1,902 (68.2%)	885 (31.8%)	2,787 (100.0%)

Treatment periods with start-date undetermined, started with the first known date of the period. Treatment periods with end-date undetermined, ended with the last known date of the period

### Validations

The validation process results of the algorithm reliability showed high level of accuracy, both in treatment periods' classification as well as in the determination of the periods' start and end-dates (Table 4).

**Table 4:** Validation of algorithm results

	**#**	**% confirmed**
**Excluded clozapine treatment periods**		
Total number of periods	425	
Number of periods randomly selected for validation (%)	90 (21.2%)	
Number of confirmed periods^a^	76	84.4%
**Clozapine treatment periods with identified start-date**		
Total number of periods	1,902	
Number of periods randomly selected for validation (%)	211 (11.1%)	
Number of confirmed periods	210	99.5%
Start-date accuracy^b^		
Highly accurate (range of up to 15 days)	202	96.2%
Accurate (range of 15–30 days)	3	1.4%
Partially accurate (range of 30–60 days)	2	1.0%
Inaccurate (range of more than 60 days)	3	1.4%
End-date accuracy^b^		
Highly accurate (range of up to 15 days)	67	71.3%
Accurate (range of 15–30 days)	13	13.8%
Partially accurate (range of 30–60 days)	13	13.8%
Inaccurate (range of more than 60 days)	1	1.1%
No end-date to detect^c^	116 (55.2%)	
**Clozapine treatment periods with undetermined start-date**		
Total number of periods	885	
Number of periods randomly selected for validation (%)	114 (12.9%)	
Number of confirmed periods	114	100%

^a^Confirmed excluded periods are treatment periods found to be "empty", meaning no clozapine was prescribed or total length of treatment was shorter than 7 days

^b^Validated for periods with identified start-date only

^c^End-date could not be detected as patients were either transferred outside of SLaM or reached the end of the study observation window

## Discussion

This study describes the development process and implementation of an algorithm designed to identify clozapine treatment dates which can be used by researchers when conducting clozapine observational studies. By combining clinical experience with informatics expertise, we were able to create a complex algorithm relying on multiple datasets, each of which had severe limitations as a standalone source of data, but when judiciously combined, yielded highly accurate results.

The final database, which consisted of 2,787 clozapine treatment periods, can serve as an important resource for clozapine studies exploring its efficacy, safety, adherence, and other research area, which may aid to increase clozapine utilization and to prevent redundant clozapine cessation. The validation and verification process yielded very good results, showing that the carefully, specifically designed automated algorithm was successful in spotting "false" treatment periods, and was able to yield good accuracy in determining the start and stop-dates of each period.

It is common for real-world databases to suffer from missing, redundant, and falsely entered information. Errors are bound to occur, especially when the users contributing to this database are both numerous and heterogeneous in professional background (clinical, administrative, etc.). Prolonged development and implementation processes may further contribute to erroneous entries, as time-based changes yield non-uniform records. The algorithm presented in this study attempts to use both clinical insight as well as data-analysis procedures to overcome as many of these errors as possible.

The authors present this study as an example of what can be achieved through the multidisciplinary process of the algorithm creation, consisting of a continuous joint discussion between informaticians and clinicians. While the latter had brought their clinical expertise along with insight into the reality of clinical practice, the informaticians could translate those insights into the structure of the database and relay the numerous problems back to the clinicians for further exploration and feedback. Both the coding itself and the clinical deliberations were conducted collaboratively throughout the process.

### Limitations

The main limitation of the methodology stems from missing data. The start-date was indeterminable for over 30% of the treatments. Despite the interplay between the three datasets and the encouraging validation results, missing data was present in all datasets, leading to misclassification or inaccurate dating. As dates might have shifted and valid treatment periods could have been excluded (or not recorded), epidemiological data should be interpreted with caution.

An additional limitation is the possible truncation of treatment periods. It was the authors’ intention to avoid over-merging (joining two separate treatment periods into one), thus risking over-truncation. The authors felt that future studies that might rely on this database, would preferentially accept possible redundant truncation and missing data, as opposed to falsely assumed data. A simple example is patients who were transferred to other hospital Trusts for periods of time between periods of SLaM care, so a break was recorded in the treatment period, often without available documentation whether clozapine treatment continued seamlessly, or was halted and then renewed. Another prominent example is treatment periods with undetermined start-date, which are recorded shortly after a previous clozapine treatment. One option to consider would be that the truncation is a mere technical fault, and that the two periods are actually a continuum. A second option would be that the subsequent treatment is a new clozapine period, for which the algorithm failed to identify a start-date. When the gap exceeded 2–3 months, a third explanation of a clozapine initiation in a different trust is also possible. To avoid redundant merging, it was decided not to merge these periods with the previous period. Many heuristics were examined in order to differentiate and decipher those instances, but none were proven to be sufficiently reliable. Even though the start-date can sometimes be ascertained over a relatively narrow timeframe (as it must occur after the previous end-date), it was decided to leave those labelled as "undetermined start-date" to mark the uncertainty arising. In several cases, for example, it was found that the previous treatment period had ended somewhat earlier than the attributed end-date, making the in-between-periods gap important enough to address these two treatment periods as separate. For example, a patient that was lost to follow-up and stopped taking clozapine might present to the emergency department, and a registration of "interrupted" status would then be recorded, along with the same day record of "new treatment" due to admission to a psychiatric ward.

Contrary to possible over-truncation of treatment periods, some treatment periods may contain several cycles of titration and re-titration. During validation, several incidents in which monthly blood tests returned to weekly pattern were preserved, suggesting clozapine cessation and re-titration. Sampling clinical notes in those dates showed that some of these cases are indeed interruptions or even "micro-interruptions" (while other may be a response to an abnormal blood test, does escalation, etc.). Future improvements of the algorithms can be devised to detect pattern changes, and better outline these conjoined treatment periods.

The validation process showed that 15% of the excluded treatments were false negative, meaning that clozapine was in fact administered in those periods. Though not many, these windows may still contain valuable information. The treatment periods mislabelled and mistakenly excluded were either too short for blood-pattern recognition or were commenced during the early years of the existence of ZTAS and CRIS early, when data was often not recorded properly.

It should be noted that while the database generated by the algorithm can be applied to various aspects of clozapine research, it is not necessarily representative of all clozapine users. The database includes all patients prescribed clozapine within SLaM, which may differ demographically from populations in other regions of the UK or worldwide. However, the significant over-representation of male patients in our sample has been observed in the UK with similar proportions and in other parts of the world, albeit to a lesser extent [23, 24].We believe the implications of our study are twofold. The first, more concrete outcome is the creation of a robust clinical database that can facilitate further observational studies on clozapine. The second, albeit currently less tangible, result is the potential to adapt the heuristics and methodology of our algorithm for use in other large psychiatric services to produce additional clozapine databases. However, achieving this goal necessitates significant adaptations due to regulatory differences between countries [25], such as variations in blood monitoring frequencies and protocols, as well as differences in database structures, the availability of additional reliable datasets (such as dosage information), or the absence of datasets utilized in our methodology. A future area of interest is the development of an algorithm to identify clozapine-induced adverse effects, particularly in relation to clozapine doses. The development of such a tool can have diverse benefits for ensuring patient safety.

## Conclusions

This paper describes a highly tailored algorithm developed through close collaboration between clinicians and data scientists. The combined expertise in clinical practices, particularly regarding the medication of interest, along with proficiency in data acquisition and analysis, facilitated the creation of an extensive database comprising clozapine treatment periods. Consequently, this paper presents two applicable products. Firstly, it introduces the described validated clozapine treatment database. Secondly, it presents a validated methodology for compiling clozapine treatment databases, which can be adapted to other large routine clinical databases, in the UK or globally, with necessary modifications to accommodate varying dispensing and blood monitoring regulations. These databases, as SLaM's clozapine database, may serve as a useful tool for researchers through two approaches. Firstly, it may serve as a platform for large dataset queries, for instance when exploring comparisons with other antipsychotics. Secondly, it may serve as a portal to specific sub-populations, which are often challenging to investigate, enabling the study of rare phenomena or clozapine-specific events. future endeavours should aim to include more detailed data, such as dosage information and adverse events.

### Supplementary Information

Supplementary material 1.

## Data Availability

The data used in this study is available in the CRIS system, as well as the database created by this study. However, CRIS data is available to researchers at SLaM only, due to patients' confidentiality. Access to it require authorization from SLaM BRC (Biomedical Research Center).
