# Supplementary material for: Developing a validated methodology for identifying clozapine treatment periods in electronic health records
Source: BMC Psychiatry. 2024 Aug 27;24:584. doi: 10.1186/s12888-024-06022-5 (PMC11351314; doi:10.1186/s12888-024-06022-5)
Supplement: Supplementary file 1 — Supplementary material 1. [file 12888_2024_6022_MOESM1_ESM.docx]

**Supplementary**

S1. Blood pattern classification

As described, blood tests were analysed to detect one of four patterns, that can assist in verifying the authenticity of the treatment period and the reliability of its start-date.

1. *Sustained weekly pattern* – This patten can serve as a reliable indication for both "real" clozapine treatment period (as opposed to "empty treatment" period in which clozapine was intended but eventually not prescribed) and for the actual start-date of clozapine initiation. To determine presence of a weekly pattern, we ascertained the following conditions:
   1. The ratio of blood tests per week during the first 18 weeks (or less, if the tentative treatment period was shorter than 18 weeks) was greater than 0.75 per week (allowing possible short delays and occasional misses).
   2. The longest gap between two blood tests during the first 18 weeks (or the length of clozapine treatment period, if shorter) did not exceed 15 days (as a "true clozapine initiation" is mandated to be accompanied by weekly blood tests).
   3. The pattern persists for a minimum of 5 weeks.
2. *Short weekly pattern* – This pattern can also serve as a reliable indication for the actual start-date of clozapine initiation, but it is not long enough to validate actual treatment. A common example might be a patient that was due to start clozapine but refused to commence. In such cases, a start-signal would exist, as well as repeated blood tests for a few weeks, without actual administration. Therefore, it was important to isolate these short weekly patterns from the "sustained weekly" pattern. However, these short patterns can suggest a "real" clozapine treatment, that ended early from various reasons, such as patients' preferences or adverse events. The criteria for the assignment of this pattern were the same as for the sustain weekly pattern, aside from criterion C.
3. *Monthly pattern* – This pattern does not indicate the exact date of clozapine initiation. However, it can verify that the patient was not just registered on ZTAS but was indeed, at a very high likelihood, receiving clozapine treatment. This patten was especially important to those patients who started clozapine before the initiation of CRIS and ZTAS or transferring into SLaM from another trust while already on clozapine. The assignment of this pattern required the following conditions:
   1. A tentative treatment period of at least 6 months duration.
   2. During those months, the ratio of blood tests per month was over 0.75 (allowing possible short delays and occasional misses).
   3. The maximal gap between 2 blood tests was less than 65 days (accounting for a possible missing entry or a missing blood test).

Due to technical issues in ZTAS, we were aware that some patients' blood tests monitoring was not recorded for several years, usually up to 2012-3, but become systematically reported later. These cases would violate criteria B. for example, a patient that start his clozapine treatment in 2012, and had a monthly pattern from 2016 onwards, would not have any blood tests recorded for 4 years, so the ratio of blood tests per month would be lower than 0.75. For these patients, we devised an alternative set of criteria to establish monthly pattern:

1. There was at least 1-year gap between the start-signal and the first blood test.
2. The maximal gap between 2 blood tests was less than 65 days (accounting for a possible missing entry or a missing blood test).
3. The ratio of blood tests was over 0.75 per month, calculated only for the period starting at the first blood test.
4. *No pattern* – clozapine treatment periods that did not fall into any of the three previous patterns.

S2. Refinement rules

1. Amending the start-point - If the first blood test which is not defined as baseline (required for ZTAS registration and precedes actual clozapine administration) is performed over 10 days following a start-signal, the treatment period starting point was updated to the date of the first blood test. This was done due to possible delays when starting clozapine (often, patients' resistance or a problem arising from the baseline blood count). However, this update was only done in treatment periods with an identified start-date. For example, when a patient "started" a treatment period already on a monthly pattern, this change would be redundant, as this treatment would be defined as one with an undetermined start-date (as no patient would be started on clozapine with monthly monitoring). However, a delay of 20 days between a start-signal and first blood test will create a false starting point, and a more accurate one can be determined by the first blood test.
2. Amending the start-point, second iteration – A start-signal along with an adjacent blood test greatly increase the likelihood of a genuine treatment onset. However, at times the initial blood tests are not done weekly, especially when there is hesitation whether to start clozapine, either from clinicians or service users. Those cases violated the "weekly pattern" identification, and thus labelled as "no pattern" and subsequently as having an undetermined start-date. To overcome this problem, we designed the algorithm to examine the gaps between the three initial blood tests of each treatment period with undetermined start-date. In cases where one of the two initial gaps were violating the 15-day maximum gap rule (and the following gaps did not), the algorithm checked if the pattern following that "violating" gap is in line with a sustained weekly pattern. If so, the start-date of the treatment was updated to that of the first blood test (of those initial three) starting the weekly pattern, and the period was re-labelled as one with an identified start-date. If not, the period remained without a substantiated start-date, and the start-date did not change.
3. Amending the end-point - If the last blood test within a treatment period (the one preceding a stop-signal) was too far apart from the stop-signal, the end-date of the period was set according to the last blood test. This rule was devised as some patients were lost to follow-up, moved to other regions and even countries, and while their status was not updated, they did not continue their clozapine treatment. The length triggering this change of end-date relied on the duration of the treatment, and its type. In cases of treatment periods with identified start-date that lasted less than a year, the allowed gap was 21 days (as no clozapine could be dispensed without blood testing after such a period). In these treatments, the new end-date was the last blood test + 14 days. If any treatment lasted over a year, when the gap between the last blood test and the end-signal was greater than 45 days, the end-date assigned was the date of the last blood test + 30 days. For treatment periods with no pattern where the first weeks had a sustained-like pattern and the duration of the treatment was less than a year, the allowed gap was 12 days, and the end-date was defined as the date of last blood test + 7 days. This was chosen as a conservative number, accounting for a possible weekly blood test regimen required.
4. Clozapine treatment periods with shorter initiation periods - periods that are not the patient's first treatment, following a previous period separated by a short interruption (marked by an "interrupted" end-status in the database) may start with only a short weekly pattern, quickly turning into a monthly pattern. This "interruption protocol" is common as the patient quickly returns to the previous dose given. As such, those treatment periods that had a short weekly pattern of at least 5 weeks (as defined earlier), were re-labelled as treatment periods with an identified start-date.
5. Illogical gaps - As treatment periods recorded at the early stages of CRIS and ZTAS endured more errors of omission, these "pre-status" treatments were scanned to detect illogical gaps between blood test, if those were recorded. When a gap of over 75 days was identified, the pre-status period was truncated into 2 or more new different treatment periods. These were than analysed and categorized by the same heuristics described for non "pre-status" treatments.

S3. Merging Rules

| **Following period**  **Preceding period** | **Treatment periods with identified start-date** | **Treatment periods with undetermined start-date** | **Unsubstantiated treatment periods** |
| --- | --- | --- | --- |
| **Treatment periods with identified start-date** | Merge if preceding TTL<120 days and preceding is "pre-status" and GBT<15 days | Merge if preceding end-status is "pre-status" and GBT<60 days  Merge if GBT<7 days and following is not PER5 and following pattern is not "short-weekly"  Merge if GBT<45 and following is not PER5 and preceding end-status is "interrupted" and former pattern is "monthly" | No merging |
| **Treatment periods with undetermined start-date** | No merging |  |  |
| **Unsubstantiated Treatment periods** | No merging | Merge if former TTL<180 days and GBT<45 days and former is "pre-status" and former NBR>2  Merge if former TTL<180 days and GBT<45 days and former is not "pre-status" and former end-status is not "non-rechallengeable" and PSN>1 |  |

GBT – Gap Between clozapine Treatment periods; TTL – Total Treatment period Length; NBR – Number of Blood test Records; PSN – Patient's treatment period Serial Number; PER5 – 5 first weeks shows a solid weekly pattern.
